# Entropy-Based Classification of Elementary Cellular Automata under Asynchronous Updating: An Experimental Study

**DOI:** 10.3390/e23020209

**Published:** 2021-02-08

**Authors:** Qin Lei, Jia Lee, Xin Huang, Shuji Kawasaki

**Affiliations:** 1 College of Computer Science, Chongqing University, Chongqing 400044, China; 201814021027@cqu.edu.cn (Q.L.); hx69@cqu.edu.cn (X.H.); 2 Chong Key Laboratory of Software Theory and Technology, Chongqing 400044, China; 3 Information Center, State Bureau for Letters and Calls, Beijing 100017, China; 4 Faculty of Science and Engineering, Iwate University, Morioka, Iwate 020-8550, Japan; shuji@iwate-u.ac.jp

**Keywords:** asynchronous cellular automata, classification, elementary cellular automata, robustness, uncertainty, entropy

## Abstract

Classification of asynchronous elementary cellular automata (AECAs) was explored in the first place by Fates et al. (Complex Systems, 2004) who employed the asymptotic density of cells as a key metric to measure their robustness to stochastic transitions. Unfortunately, the asymptotic density seems unable to distinguish the robustnesses of all AECAs. In this paper, we put forward a method that goes one step further via adopting a metric entropy (Martin, Complex Systems, 2000), with the aim of measuring the asymptotic mean entropy of local pattern distribution in the cell space of any AECA. Numerical experiments demonstrate that such an entropy-based measure can actually facilitate a complete classification of the robustnesses of all AECA models, even when all local patterns are restricted to length 1. To gain more insights into the complexity concerning the forward evolution of all AECAs, we consider another entropy defined in the form of Kolmogorov–Sinai entropy and conduct preliminary experiments on classifying their uncertainties measured in terms of the proposed entropy. The results reveal that AECAs with low uncertainty tend to converge remarkably faster than models with high uncertainty.

## 1. Introduction

A cellular automaton (CA) is a discrete dynamical system consisting of a huge number of locally interconnected cells [1]. Each cell serves as a finite automaton that interacts with its neighbors to change the state. In particular, despite the simple interactions between cells at local level, the inherent parallelism of CAs allows the emergence of complex behavior at global level, and makes them a suitable model for parallel computing and simulations of natural complex phenomena [2].

Conventional CA models are deterministic and synchronous, in which all cells need to acquire all states of their neighbors simultaneously and undergo state transitions simultaneously at every discrete time step [3]. Wolfram [2] was the first to categorize all elementary cellular automata (ECAs) into four classes based on the complexities of their global behavior evolving from random configurations, in which the class III and IV models exhibit chaotic-like behavior and are widely considered to hold potential for universal computation [4]. More classifications of the synchronous ECAs according to different measurements have been done [5], and it was claimed [6] that even the simplest class I ECAs might offer the chance to carry out complex computations.

The strict simultaneity and determinism of synchronous CAs, however, seem not applicable in many complex natural scenarios where noises or perturbations prevail. Ingerson and Buvel [7] initially questioned the perfect synchrony hypothesis by changing the deterministic transition function of ECAs to random probability function. Especially, they observed that the updating scheme plays a fundamental role in producing the global behavior of the ECAs.

In contrast to synchronous models, asynchronous cellular automata (ACAs) allow each cell to be updated at random times and independently from other cells. Fates and Morvan [8] explored in the first place the robustness of ECAs to asynchronous transitions of cells. They carried out experiments on ECAs after clarifying the concept of robustness for ACAs [8], with an aim to testify whether the application of a small change in the way the transitions are performed leads to brutal changes of the global behavior. To this end, they examined the effect of stochastic updating on global behavior and classified the ECAs roughly into four qualitative sets according to their responses to various probabilities, including models that exhibit (a) continuous variation of the behavior (e.g., ECA 232), (b) discontinuity around probability 1 (e.g., ECA 110), (c) phase transition (e.g., ECA 50) and (d) non-regular behavior (e.g., ECA 184), respectively [3].

The above classification was accomplished through the use of an approximation of the asymptotic density as a key parameter to estimate the changes of the global behavior of ECAs quantitatively. An asymptotic density refers to the density that would be reached by an ECA consisting of an infinite number of cells, after evolving an infinite number of time steps. This parameter was considered as a first means to detect changes in the behavior, whereby a strong variation of the asymptotic density reveals that the system has undergone a transformation, whereas an absence of variation does not necessarily imply that the system remains stable [3]. However, the density-based classification seems incomplete because some ECAs failed to be classified into any of the four qualitative sets.

In order to achieve a complete classification of all ECAs and gain more insights into their dynamics, this paper proposes a novel parameter to measure the robustness of these models against stochastic transitions of cells. Our parameter depends on the distribution of local binary patterns of certain lengths in the cell space of an AECA, rather than simply the ratio of cells in state 1. To this end, we adopt a metric entropy [9] which was originally proposed to confirm the underlying dynamics of synchronous ECAs in accordance with the Wolfram’s empirical classification [2]. In Boltzmann’s definition, entropy is a measure of the number of possible microscopic states of a system in thermodynamic equilibrium, consistent with its macroscopic thermodynamic properties. Shannon [10] introduced the concept of information entropy to measure the uncertainty in information, which is the average rate at which information is produced by a stochastic source of data. Regardless of thermodynamics or information theory, the definition of entropy is identical that describes a function between microscopic and macroscopic states in complex systems.

Specifically, the metric entropy is to estimate the average quantity of information associated with the distributions of all binary patterns in the space-time diagrams [9]. Based on the metric entropy, we conduct experiments on AECAs to estimate the robustness in response to stochastic updating with various probabilities. The results show that all AECAs can possibly be classified into one of four qualitative sets according to their robustnesses, even when all binary patterns are restricted to length 1. Moreover, for synchronous ECAs, topological entropy and Kolmogorov–Sinai (KS) entropy have been defined [11,12], for the sake of measuring quantitively the uncertainty of the evolutions of deterministic dynamical systems, provided with incomplete description of initial conditions [13,14]. For AECAs, on the other hand, their global behavior may not only be sensitive to the initial configurations, but also be seriously affected by the probability controlling the state transitions of each cell at any time. This motives us to define another entropy in the form of the KS entropy, for the purpose to challenge the uncertainty measure problem of AECAs. Numerical experiments will be done to compute the entropies of each AECA model, which allow a preliminary classification of them in accordance with their estimated uncertainties.

This paper is organized as follows: Section 2 provides the definitions of AECAs and two types of entropies. Section 3 defines the protocol required for experiments and analyzes the limits of the protocol. Section 4 provides the classification of AECAs based on metric entropy, and discusses their robustness. Section 5 shows a preliminary classification of AECAs according to uncertainties measured by KS entropy. This paper finishes with conclusion given in Section 6.

## 2. Basic Definitions

### 2.1. Cellular Automata

Let Z be the set of all integers. A Cellular Automaton (CA) is defined by $\left(Z^{d},N,Q,f\right)$, where $Z^{d}$ represents *d*-dimensional array of cells (d>0). *Q* is a finite set of states (Q≠∅) and $N\subset Z^{d}$ is a finite set called neighborhood index. $f:Q^{\left|N\right|}\to Q$ is a local transition function. In addition, a configuration $x^{t}$ at time step t≥0 is a mapping $x^{t}:\;Z^{d}\to Q$ which assigns a certain state in *Q* to every cell in the cell space.

Assume $N=\left\{n_{0},n_{1},...,n_{k}\right\}$ with k=|N|−1. Synchronous CAs require all cells to undergo state transitions simultaneously at each discrete time steps in accordance with a global transition function *F*, such that for any t≥0, $x^{t+1}=F\left(x^{t}\right)$ and

$$\forall c\in Z^{d}:\;x^{t+1}\left(c\right)=F\left(x^{t}\right)\left(c\right)=f\left(x^{t}\left(c+n_{0}\right),x^{t}\left(c+n_{1}\right),\cdots ,x^{t}\left(x+n_{k}\right)\right).$$

Unlike synchronous models, asynchronous cellular automata allow their cells to update the states independently at random times. In general, there are two kinds of asynchronous updating schemes for ACAs [3,7], described as follows.

- fully asynchronous updating: At each time step, the local rule is applied to only one cell, chosen uniformly at random among the set of cells.
- α-asynchronous updating: At each time step, each cell has a given probability α to apply the rule and a probability 1−α to stay in the same state. The parameter α is called the synchrony rate.

The fully asynchronous updating scheme is surely a valid hypothesis for some particular contexts (e.g., a radioactive disintegration) [3]. But in the field of communication and distributed computing, some synchrony between agents needs to be assumed [15], and the case of fully asynchronous updating rarely happen. Moreover, unlike the probability cellular automata [16] which assign probabilities to each state that a cell may change to at every time step, the α-asynchronous updating scheme provides a probability to control the updating rates of each cell at any time. This paper adopts the α-asynchronous updating scheme to iterate the evolutions of ACAs, by which the global transition function becomes $\forall t\in N,\forall c\in L,N=\{n_{0},n_{1},...,n_{k}\}:$

(1) $$x^{t+1}\left(c\right)=F_{\Delta }\left(x^{t}\left(c\right)\right)=\left\{\begin{array}{cc} f(x^{t}\left(c+n_{0}\right),x^{t}\left(c+n_{1}\right),\ldots ,x^{t}\left(c+n_{k}\right)) & if\;c\in \Delta (t)\;\\ x^{t}\left(c\right) & otherwise \end{array}\right.$$

where Δ(t):N→P(L) is a selection function which gives for time *t* the subset of cells to be updated, where each cell has a probability α to be selected [17].

### 2.2. Asynchronous Elementary Cellular Automata

An Elementary Cellular Automaton (ECA) is the simplest one-dimensional cellular automaton in which each cell takes a binary state and only access its nearest neighbors to the left and right at all times. Numerical experiments, in general, assume the cell space of an ECA consisting of a finite number of cells under periodic boundary condition, i.e., a ring. Thus, it turns out to be possible to define an ECA as (L,N,{0,1},f) where:

- L=Z/LZ is an one-dimensional ring of length *L*,
- N={−1,0,+1},
- $f:{\left\{0,1\right\}}^{3}\to \left\{0,1\right\}$ is the local transition function.

In addition, every ECA can be uniquely labelled by a decimal code *m* where $m=f\left(0,0,0\right)\cdot 2^{0}+f\left(0,0,1\right)\cdot 2^{1}+\cdots +f\left(1,1,1\right)\cdot 2^{7}$. As a result, there are 256 ECA models (rule spaces) in total. Due to left/right reflexion and 0/1 complementarity, it is possible to narrow down the 256 ECA rule spaces to 88 classes, each represented by a function with the smallest code [18]. In later experiments, we merely consider 88 minimal representative ECA rules.

In this paper, asynchronous ECAs (AECAs) employ the α-updating scheme to iterate the state transitions of cells, with the synchrony rate ranging from a perfect synchronism (α=1) to the limiting case of full asynchronism (α→0). In addition to the synchrony rate, the density of initial configuration $d_{ini}$ also serves as an essential variable to estimate the robustness of an AECA, that is, whether the model will totally or partially resist the perturbation of its updating scheme. To this end, we define the quantity of information as a key parameter below rather than the density of cells in state 1 in any configuration evolving from the initial configuration.

### 2.3. Metric Entropy

Let $S_{k}$ be the set of all binary strings of length k≥1, i.e., $S_{k}={\left\{0,1\right\}}^{k}$. Moreover, let (L,N,{0,1},f) be an AECA with L=Z/LZ, and suppose $x^{t}$ is the configuration at time step t≥0 evolving from an initial configuration $x^{0}$, i.e., $x^{t}=F_{\Delta }^{t}\left(x^{0}\right)$ and $x^{t}\in {\left\{0,1\right\}}^{L}$. For each pattern $s\in S_{k}$, let $\#(s,x^{t})$ depict the number of occurrences of the string *s* in configuration $x^{t}$, that is,

$$\#\left(s,x^{t}\right)=\left|\left\{i\;|\;0\leq i<L\wedge s=x^{t}\left(i\right)x^{t}\left(i_{1}\right)\cdots x^{t}\left(i_{k-1}\right)\right\}\right|$$

where $\forall j\in \left\{1,\cdots ,k-1\right\}:\;i_{j}=\left(i+j\right)\;\mathrm{mod}\;L$. In this case, define a function $p_{k}^{t}:\;S_{k}\to \left[0,1\right]$ such that

(2) $$\forall s\in S_{k}:p_{k}^{t}\left(s\right)=\frac{\#(s,x^{t})}{L}.$$

For example, suppose $x^{t}=0011011101$. Obviously, if k=1 and $S_{1}=\left\{0,1\right\}$, $p_{1}^{t}\left(1\right)=0.6$ and $p_{1}^{t}\left(0\right)=0.4$ coincide with the densities of cells in state 1 and 0 in $x^{t}$, respectively. If k=2 such that $S_{2}=\left\{00,01,10,11\right\}$, we obtain $p_{2}^{t}\left(00\right)=0.1$ and $p_{2}^{t}\left(01\right)=p_{2}^{t}\left(10\right)=p_{2}^{t}\left(11\right)=0.3$.

Because $\sum_{s\in S_{k}}p_{k}^{t}\left(s\right)=1$, it turns out to be possible to construct a probability space $\left(S_{k},2^{S_{k}},P^{t}\right)$ over the configuration $x^{t}$, where $P^{t}:\;2^{S_{k}}\to \left[0,1\right]$ is a probability measure function that satisfies $\forall A\subseteq S_{k}:\;P^{t}\left(A\right)=\sum_{a\in A}p_{k}^{t}\left(a\right)$. This allows to estimate the entropy $M_{k}\left(x^{t}\right)$ of local patterns (of length *k*) distributing in configuration $x^{t}$ as follows:

(3) $$M_{k}\left(x^{t}\right)=-\sum_{s\in S_{k}}p_{k}^{t}\left(s\right)\log\left(p_{k}^{t}\left(s\right)\right).$$

Assume the AECA (L,N,{0,1},f) iterates the global transitions for *t* time steps (t≥0). Then the metric entropy ${\bar{M}}_{k}$ of the AECA can be estimated by using the averaged entropy for each configuration:

(4) $${\bar{M}}_{k}=\lim_{L\to \infty }\lim_{t\to \infty }\frac{\sum_{j=0}^{t}M_{k}\left(x^{j}\right)}{t}.$$

Since an AECA defines a system with continuous changes in α and $d_{ini}$, ${\bar{M}}_{k}$ can be regarded as a continuous function of α and $d_{ini}$, that is, ${\bar{M}}_{k}\left(d_{ini},\alpha \right)$. Especially, the calculation of ${\bar{M}}_{k}$ is based on the logarithmic method of entropy, and the stop time will affect the value of ${\bar{M}}_{k}$ sensitively. Specifically, it is expected an approximation of the asymptotic ${\bar{M}}_{k}$, that is, the value of the ${\bar{M}}_{k}$ that would be reached by an infinite-size system with an infinite simulation time [8,19].

### 2.4. Kolmogorov–Sinai Entropy for AECAs

Let (L,N,{0,1},f) be an AECA where L=Z/LZ, and let $C_{L}$ be the set of all configurations of the AECA, i.e., $C_{L}={\left\{0,1\right\}}^{L}$. Assume $x^{0}\in C_{L}$ is an initial configuration. Evolving from the configuration $x_{0}$ by global transitions of cells gives rise to a sequence of configurations: $x^{0},x^{1},x^{2},\cdots ,x^{t-1},x^{t},\cdots$ such that $\forall t>0:\;x^{t}=F_{\Delta }\left(x^{t-1}\right)$. Due to the α-asynchronism, this sequence is deterministic and unique when α=1, whereas it becomes a stochastic process if α<1.

Assume n>0 and $x^{0},x^{1},\cdots ,x^{n}$ is a sequence of configurations evolving from initial configuration $x^{0}$. For simplicity, let $\Gamma_{n}$ denote the sequence $<x^{0},\cdots ,x^{j},\cdots ,x^{n}>$, which actually constitutes the space-time diagram of the AECA. For any $c\in C_{L}$, let $\#(c,\Gamma_{n})$ denote the number of occurrences of configuration *c* in the sequence $\Gamma_{n}$, whereby

$$\#\left(c,\Gamma_{n}\right)=\left|\left\{j\;|\;0\leq j\leq n\wedge c=x^{j}\right\}\right|.$$

Thus, it is possible to define a probability measure $\mu_{n}:\;C_{L}\to \left[0,1\right]$ over $\Gamma_{n}$ such that

$$\forall c\in C_{L}:\;\mu_{n}\left(c\right)=\frac{\#(c,\Gamma_{n})}{n+1}.$$

Because $\sum_{c\in C_{L}}\mu_{n}\left(c\right)=1$, the measure space $\left(C_{L},2^{C_{L}},P^{n}\right)$ turns out to be a probability space, in which the measure function $P^{n}:\;2^{C_{L}}\to \left[0,1\right]$ satisfies $\forall B\subseteq C_{L}:\;P^{n}\left(B\right)=\sum_{b\in B}\mu_{n}\left(b\right)$.

As a result, the probability space $\left(C_{L},2^{C_{L}},P^{n}\right)$ offers the opportunity to calculate the entropy $H_{n}$ of the AECA over the space-time diagram $\Gamma_{n}$, by adopting the Shannon’s entropy equation:

(5) $$H_{n}=-\sum_{c\in C_{L}}\mu_{n}\left(c\right)\log\left(\mu_{n}\left(c\right)\right).$$

The value of $H_{n}$ will reach the maximum $\log(|C_{L}|)$ when each configuration in $C_{L}$ appears in the diagram $\Gamma_{n}$ with equal frequency. The entropy $H_{n}$ may possibly be used to measure the uncertainty of the AECA. Generally speaking, the larger the $H_{n}$, the larger the uncertainty of the system, and vice versa. In addition, similar to the metric entropy ${\bar{M}}_{k}$, for an AECA system, $H_{n}$ is also a continuous function of α and $d_{ini}$, that is, $H_{n}\left(d_{ini},\alpha \right)$. Thus, it makes sense to define a KS entropy $H_{ks}$ in the following way [20]:

(6) $$H_{ks}=\underset{d_{ini},\alpha }{\sup}\lim_{L\to \infty }\lim_{n\to \infty }\left(H_{n}\left(d_{ini},\alpha \right)\right).$$

It is worth noting that it is difficult to reach the limit in our experiments. We will use a larger value to replace infinity, that is, we will use $\max H_{n}$ to replace $\sup H_{n}$.

## 3. Experiment Protocol

In this section, we will introduce two classification protocols for metric entropy ${\bar{M}}_{k}$ in Equation (4) and entropy $H_{n}$ in Equation (5), both of which are functions of α and $d_{ini}$. The effect of changes in α and $d_{ini}$ will be observed by a specific function *g*.

### 3.1. Stop Time of Iterations

The stopping time decides the time point to interrupt the iterations of an AECA, which usually depends on the purpose of experiments. For the sake of measuring two types of entropies during the iterations, it is natural to choose a time point, if exists, at which the evolution of the AECA converges as the stopping time.

Let (L,N,{0,1},f) be an AECA and assume $x^{t}\in {\left\{0,1\right\}}^{L}$ is a configuration at time step t≥0. The configuration $x^{t}$ is called a fixed point if no cell in it can actually change the state via the local function *f*, i.e.,

$$\forall c\in L:\;f\left(x^{t}\left(c-1\right),x^{t}\left(c\right),x^{t}\left(c+1\right)\right)=x^{t}\left(c\right).$$

Such property is independent of the updating scheme, which implies that both the synchronous and asynchronous updating methods induce the same set of fixed points [21]. Accordingly, in each experiment, we first apply the synchronous global function *F*, rather than the α-asynchronous function $F_{\Delta }$, to the current configuration $x^{t}$ such that if $F\left(x^{t}\right)=x^{t}$ then stop further iterations; otherwise, applying $F_{\Delta }$ to $x^{t}$ to obtain the configuration $x^{t+1}$ at the next time step. If the AECA does not converge, the experiment will be stopped at *N*-th iteration where N>0 is a predefined maximal number of iterations.

### 3.2. Definition of Experimental Protocol

The macroscopic measures we used are based on the statistical analysis of the two kinds of entropies, which are functions of the synchrony rate α as well as the initial density $d_{ini}$ as said before. In particular, we employ an observation function *g* (also called sampling surface [8]) to denote the variations of the entropies with α and $d_{ini}$:

(7) $$g\left(d_{ini},\alpha \right)=\frac{1}{T}\sum_{i=1}^{T}H\left(d_{ini},\alpha \right)$$

where *T* is the number of repeated trials and the function *H* stands for either the metric entropy ${\bar{M}}_{k}$ in Equation (4) or the entropy $H_{n}$ in Equation (5) The independent variables $\left(d_{ini},\alpha \right)$ are actually two hyper-parameters of an AECA. That is, the hyperparameter $d_{ini}$ is implicitly contained in $x^{0}$ which means a uniform density sampling under the parameter $d_{ini}$. Similarly, the hyperparameter α is implicit in the global transition function $F_{\Delta }$. In practical experiments, we must do a sampling by randomly choosing some initial conditions and some synchrony rates. In such a situation, let $D=\left[d_{min},d_{max}\right]\left(d_{stp}\right)$ denote the range of initial densities varying from $d_{min}$ to $d_{max}$ with step $d_{stp}$. Likewise, let $A=\left[\alpha_{min},\alpha_{max}\right]\left(\alpha_{stp}\right)$ be the set of synchrony rates that will be utilized in the experiments.

Algorithm 1 describes in detail the process of constructing the observation function $g_{{\bar{M}}_{k}}$ and $g_{H_{N}}$, where lines 9 to 11 describe the moment of convergence mentioned in Section 3.1. It outputs two sets of points $g_{{\bar{M}}_{k}}$ and $g_{H_{N}}$ corresponding to each $(d_{ini},\alpha )\in D\times A$, plotted in a 3D space in the form of a two-dimensional sampling surface [8]. Algorithm 1 mainly uses the idea of averaging to control randomness. Therefore, the setting of the value of parameters becomes critical. The specific parameters of the experiment are as follows:

- D=[0.2,0.8](0.05) and A=[0.2,1](0.05)
- The number of max generations *N* = 10,000
- The number of repeated trials T=400
- The length of cellular ring L=800

### 3.3. Protocol Limits

Like in any simulation approach, the protocol design may affect or limit the validity of the experimental results. The limitations and effectiveness of the sampling surface have been discussed by Fatès [8], here we only discuss the limitations of the numerical simulation in our experiments.

Although $g_{{\bar{M}}_{k}}$ and $g_{H_{N}}$ are calculated at the same time in Algorithm 1, in practice, simulation experiments are often performed separately. This is because if all 88 minimal representative ECA rules are simulated according to the parameter settings mentioned in Section 3.2, the time taken may become unacceptable. To compute $g_{{\bar{M}}_{k}}$, a significantly high accuracy can be obtained when using the parameter settings mentioned in Section 3.2. An acceptable accuracy can also be obtained if the parameter settings are relaxed to L=200,N=1500,T=200, which, in turn, will speed up the experiments to a large degree.

The above relaxed parameter settings, on the other hand, are unavailable to the experiments on the KS entropy $g_{H_{ks}}$. Generally speaking, the upper bound of iterations of *N* should be no less than the scale of the cell space $2^{L}$, which is essential to non-convergent ACEAs, like AECA 90. To avoid such issues, we may reduce the value of *L* to a small number (e.g., 10), but this can only be applicable to those AECAs that are not sensitive to the initial density $d_{ini}$. Another way is to specify an acceptable upper bound of iterations such as *N* = 10,000, which can possible guarantee the quantitative analysis of the experiments, and its influence on the experimental results is mainly in the morphology of the sampling surface, making the precise morphology generalized.
**Algorithm 1** Construction of sampling surfaces**Input:** The array of densities of the initial configuration, $D=\left[d_{min},d_{max}\right]\left(d_{stp}\right)$; The array of synchronous rates, $A=\left[\alpha_{min},\alpha_{max}\right]\left(\alpha_{stp}\right)$; The cell ring L with length *L*; The number of maximal iterations *N* and the number of repeated trials *T*.**Output:** Two sampling surfaces $\left(g_{{\bar{M}}_{k}},g_{H_{N}}\right)$ 1:$X=\{x:x_{d_{ini}}^{0}\in L,d_{ini}\in D\}\leftarrow$ Initialize the initial configuration array **X**, each *x* follows a uniform density sampling with density $d_{ini}$. 2:T=[1,2,...,T]. 3:**for each**α∈A**do** 4:   **for each** x∈X **do**
 5:      **for each** n∈T **do**
 6:         $space\left[0\right]=x_{d_{ini}}^{0}$
 7:         **for each** t∈[0,1,...,N] **do**
 8:            **if** $x^{t}==F\left(x^{t}\right)$ **then**
 9:               **break**10:            **end if**
11:            $x^{t+1}=F_{\Delta }\left(x^{t}\right)$
12:            $space\left[t+1\right]=x^{t+1}$
13:         **end for**14:         ${\bar{M}}_{k}\left[\alpha ,x,n\right]\leftarrow space\left[1,\cdots ,N\right]$ (Equation (4))15:         $H_{N}\left[\alpha ,x,n\right]\leftarrow space\left[1,\cdots ,N\right]$ (Equation (6))16:      **end for**17:   **end for**18:**end for**19:$g_{{\bar{M}}_{k}}={\bar{M}}_{k}.mean\left(axis=2\right)\leftarrow$ Average repeated trials20:$g_{H_{N}}=H_{N}.mean\left(axis=2\right)\leftarrow$ Average repeated trials

## 4. Classification of Robustness Based on Metric Entropy

As defined in Section 2.3, ${\bar{M}}_{k}$ is a metric entropy obtained by averaging all Shannon entropy of distribution of local patterns of length k>0 in a simulation of an AECA. Here we focus on k=1, that is, the distribution of cells in state 0 and state 1 in all configurations. For simplicity, we denote ${\bar{M}}_{1}$ by $\bar{M}$. Thus, it seems similar to the density parameter used by Fates [8] to classify most, but not all, AECA models. It is worth noting that, in addition to the difference between the experimental protocols (the main difference is the period of obtaining the parameters), the difference between entropy and density is also obvious. Density is an intuitive parameter that can easily express the characteristics of phase transition. The parameters of entropy are not so intuitive (for metric entropy $\bar{M}$, it is the average after the logarithm of the density).

**Figure 1:**
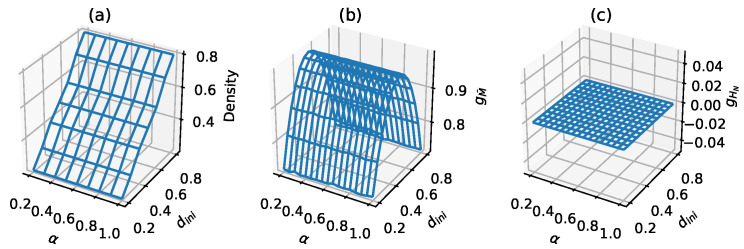
Sampling surfaces of AECA 204 under three different parameters and protocols: (**a**) density parameter (the protocols described in [8]), (**b**) metric entropy $\bar{M}$ and (**c**) entropy $H_{N}$ (the protocols described in Section 3).

For example, AECA 204 is a trivial CA model which only keeps the initial configuration unchanged regardless of the synchrony rate α. Figure 1a,b illustrate two two sampling surfaces, in which the density-based sampling surface is intuitive and linear, while the metric entropy-based surface displays a typical saddle-shape with maximum entropy occurring when state 0 and 1 are equally distributed in a configuration.

### 4.1. Results of Robustness-Based Classification

A sampling surface is usually used to describe the robustness of a dynamical system [3,8]. The sampling surface $g_{\bar{M}}$ is to observe the robustness of the system through $\bar{M}$, which is different from the density parameter, as shown in Figure 1. Thus, it allows to achieve a different classification from the density parameter. The classification criteria are as follows:

1. Qualitative analysis of sampling surface morphology, such as flat surface, continuous smooth surface, etc.
2. Quantitative numerical analysis, such as the entropy range when α is in the interval [0.2,1) and the discontinuous mutation when α=1, etc.

According to the above criteria, the AECA rule spaces based on the $g_{\bar{M}}$ can be divided into the following four classes (Table 1):

- Class 1: The sampling surface $g_{\bar{M}}$ is a high-entropy plane approximately, and discontinuous abrupt changes appear when α=1.
- Class 2: The sampling surface $g_{\bar{M}}$ is a low-entropy slope approximately, and discontinuous abrupt changes appear when α=1.
- Class 3: A continuous surface with large fluctuations in the value range, where $d_{ini}$ plays a key role in the influence of the sampling surface $g_{\bar{M}}$ than α generally.
- Class 4: A smooth continuous surface with large fluctuations in the value range, where α has a stronger influence on sampling surface $g_{\bar{M}}$ than $d_{ini}$. Most of the rules show second-order phase transition (SPT).

### 4.2. Details of Classification of Robustness

**Figure 2:**
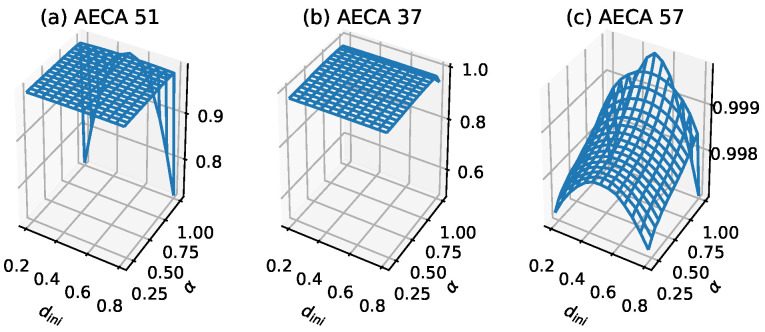
Samples of sampling surface $g_{\bar{M}}$ of Class 1: (**a**) AECA 51, (**b**) AECA 37 and (**c**) AECA 57. $g_{\bar{M}}$ is represented by the z-axis. The characteristic of the sampling surfaces of Class 1 is a high-entropy plane. Moreover, the surface in (**c**), despite its display, still approximates a plane because of the small variations in values on the z-axis.

The specific numerical characteristics of AECAs in Class 1 are as follows: $g_{\bar{M}}$ satisfies its minimum min>0.9, and it’s difference diff between the maximum and minimum satisfies diff<0.1 in the interval α=[0.2,1). According to the different performance of $g_{\bar{M}}$ at α=1, it can be divided into 2 sub-categories: (i) obvious mutation (Figure 2a), and (ii) non-obvious mutation (Figure 2b). Part of the rules in Class 1b (which are underlined in Table 1) will gradually appear continuous or discontinuous minima in several of the four corners of the sampling surface ($\left\{\left[\alpha ,d_{ini}\right]:\alpha =\left\{0.2,1\right\},d_{ini}=\left\{0.2,0.8\right\}\right\}$).

**Figure 3:**
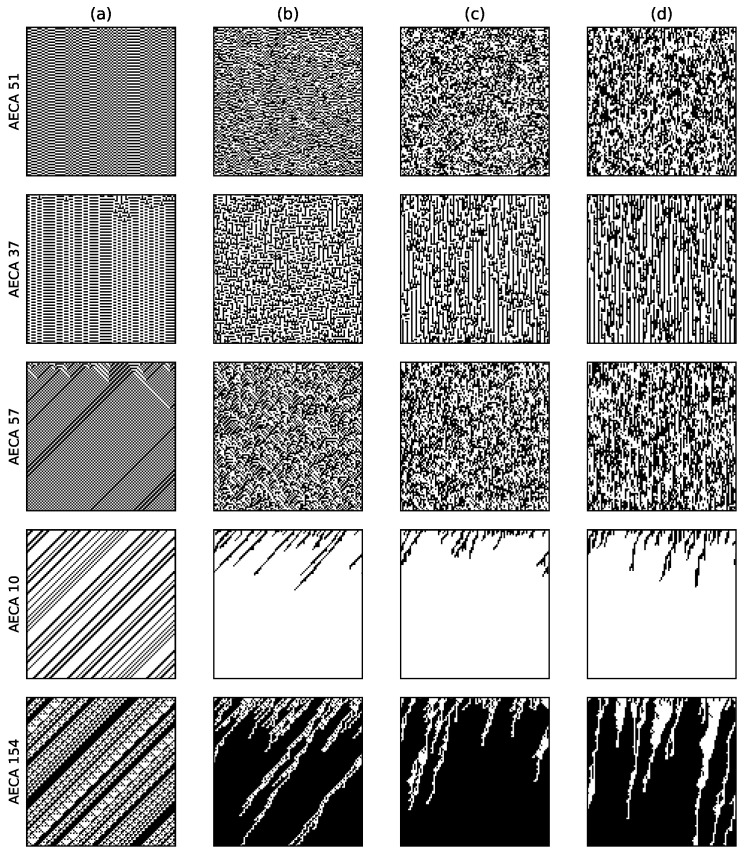
Time-space diagrams of some AECAs evolving under various synchrony rates: (**a**) α=1, (**b**) α=0.8, (**c**) α=0.5 and (**d**) α=0.3. All initial configurations are the same, with $d_{ini}=0.5$. Time flows from top to bottom.

Figure 3 shows the time-space evolution diagrams of AECA 51, 37, and 57 respectively. When α=1, they become synchronous ECAs. When α<1, these models show strong robustness due to their non-obvious change in the global behavior, although it is difficult to find the law behind. This can also be seen from the corresponding sampling surfaces in Figure 2. They are almost flat when α<1, so the change of initial configurations and α have tiny effect on the systems generally.

The specific numerical characteristics of Class 2 are as follows: $g_{\bar{M}}$ satisfies max<0.6, and 0.1<diff<0.3 in the interval α=[0.2,1). For the low-entropy slope $g_{\bar{M}}$ with α=[0.2,1), the increase of $d_{ini}$ tends to cause the surface $g_{\bar{M}}$ to increases. Especially, the more obvious the trend becomes, the higher the value of $d_{ini}$ is. Likewise, the increase of α will give rise to the decease or invariant of the $g_{\bar{M}}$. When α=1, discontinuous mutations as well as maximal value appear in the sampling surface $g_{\bar{M}}$ of all AECAs in Class 2, which may reveal a certain functional relationship with $d_{ini}$.

**Figure 4:**
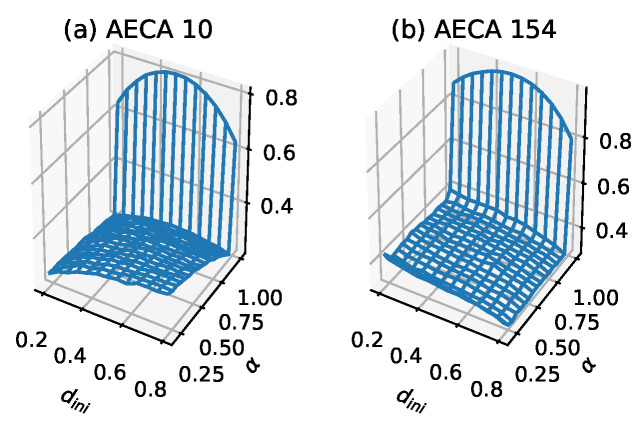
Sampling surfaces $g_{\bar{M}}$ of (**a**) AECA 10 and (**b**) AECA 154 in Class 2, in each of which a low-entropy slope with discontinuous mutation can be observed.

Figure 4a illustrates typical sampling surface of most AECAs in Class 2. Exceptions are the AECA 8 and AECA 154 (Figure 4b), of which the sampling surfaces may slightly differ in the slope direction from other models as shown in Figure 4. Nevertheless, both surfaces in Figure 4 indicate strong robustness of the corresponding AECEs, which can also be verified through the time-space diagrams of AECA 10 and AECA 154 in Figure 3 in response to the change of synchrony rate α.

**Figure 5:**
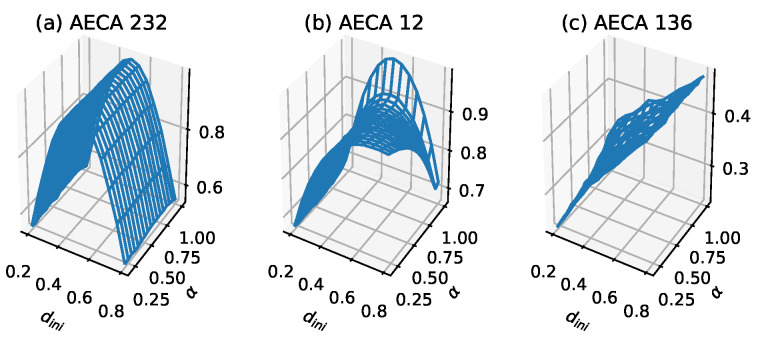
Sampling surface $g_{\bar{M}}$ of (**a**) AECA 232, (**b**) AECA 12 and (**c**) AECA 136. $g_{\bar{M}}$ in Class 3. The characteristic of these sampling surfaces is that the value range of $g_{\bar{M}}$ fluctuates substantially, that is, diff>0.35 on average.

The specific numerical characteristics of Class 3 are as follows: the sampling surface $g_{\bar{M}}$ satisfies diff>0.35 on average and $d_{ini}$ has stronger effect on $g_{\bar{M}}$ than α. There are three types of $g_{\bar{M}}$: (a) smooth arched surfaces, (b) semi-arched surfaces and (c) inclined surfaces. For (a) the smooth arch surfaces like the AECA 232 (Figure 5a) and AECA 204 (Figure 1b), the synchrony rate α has a negligible effect on the sampling surface $g_{\bar{M}}$, and thus, $g_{\bar{M}}$ can possibly be regarded as a function of $d_{ini}$ where diff>0.2 and max>0.86. In addition, for (b) the semi-arch surfaces, α has a certain influence on $g_{\bar{M}}$, and some AECAs will reveal abrupt changes in the corners of $g_{\bar{M}}$ $\left\{\left[\alpha ,d_{ini}\right]=\left[1,0.8\right]\right\}$, making the sampling surface $g_{\bar{M}}$ irregular. Especially, $g_{\bar{M}}$ will reach the maximum at α=1, as shown by the AECA 12 in Figure 5b. For (c) the inclined surfaces, the effects of α and $d_{ini}$ on $g_{\bar{M}}$ are almost the same, giving rise to a continuous inclined surface. In particular, most of the AECAs with inclined surface shows a substantial fluctuation in the range of values of $g_{\bar{M}}$ such that max<0.6 and diff>0.22, similar to the AECA 136 in Figure 5c.

**Figure 6:**
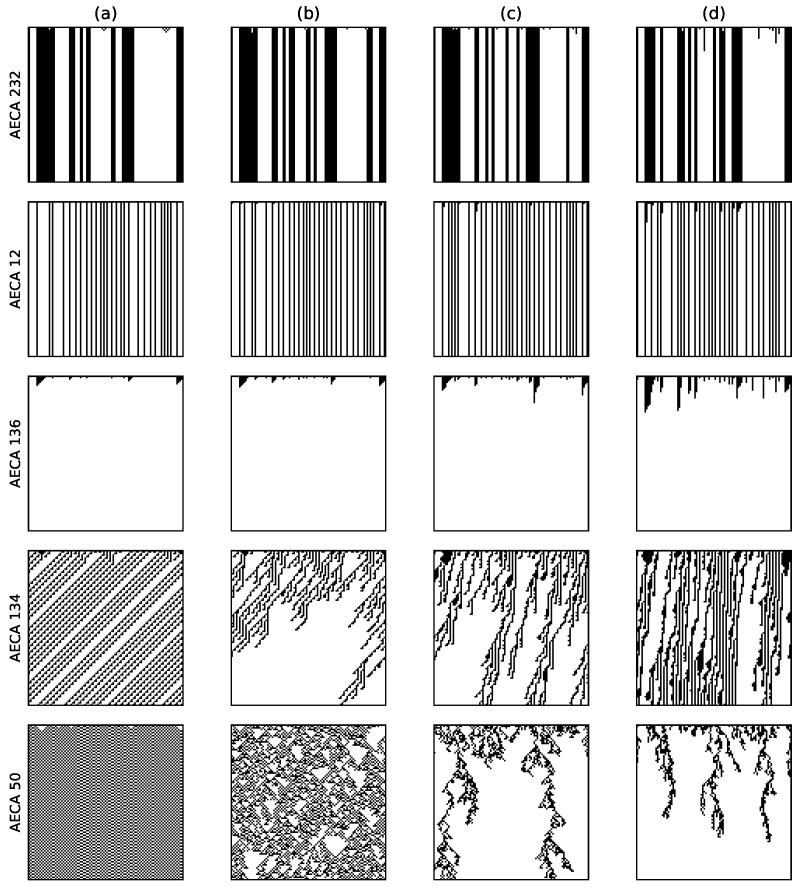
Time-space diagrams of some AECAs evolving under various synchrony rates: (**a**) α=1, (**b**) α=0.8, (**c**) α=0.5 and (**d**) α=0.3. All initial configurations are the same, with $d_{ini}=0.5$. Time in each diagram flows from top to bottom.

The characteristic of the AECAs in Class 3 is that on average, diff>0.35 in the interval [0.2,1), which indicates to a certain extent that the robustness of the rules is poor. Since most of these rules show that $d_{ini}$ is the main influencing factor of their sampling surface $g_{\bar{M}}$, the time-space diagrams of AECA 232, 12 and 136 in Figure 6 have no evident changes along with the synchrony rate α.

Except AECA 46 (underlined in Table 1), the rest of the rules of Class 4 have been proved to show second-order phase transitions (SPT), belonging to the directed percolation (DP) universality [21,22,23,24]. Thus, Class 4 is consistent with the density-based classification except the discontinuous mutation on the sampling surface at α=1 caused by the different protocols. In this case, $d_{ini}$ has almost no effect on $g_{\bar{M}}$, and $g_{\bar{M}}$ can be regarded as a function of α and satisfies diff>0.48 on average.

**Figure 7:**
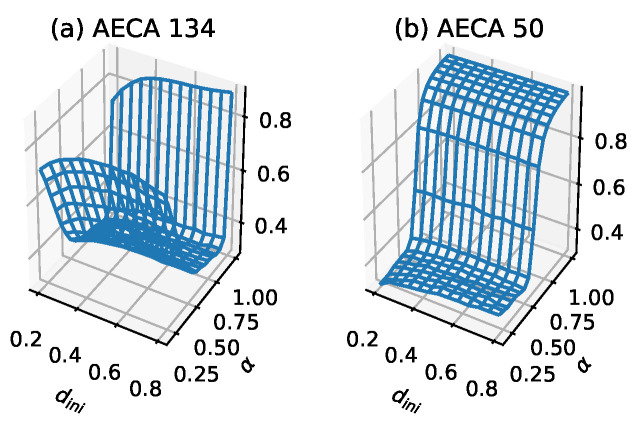
Sampling surface $g_{\bar{M}}$ of (**a**) AECA 134 and (**b**) AECA 50 in Class 4. The characteristic of the sampling surface of Class 4 is a smooth continuous surface with large fluctuations.

The AECA 134 and AECA 50 in Figure 7 belong to DP${}_{hi}$ and DP${}_{low}$, respectively [22,24]. Because the critical point of AECA 50 is around α=0.628 [24], its time-space diagrams in Figure 6 exhibits substantially difference at α=0.5 and α=0.2. On the other hand, because the AECA 134 has a critical point at α=0.082, no significant difference arises in the time-space diagrams of this rule in Figure 6.

### 4.3. Difference between Metric Entropy-Based and Density-Based Classifications

**Figure 8:**
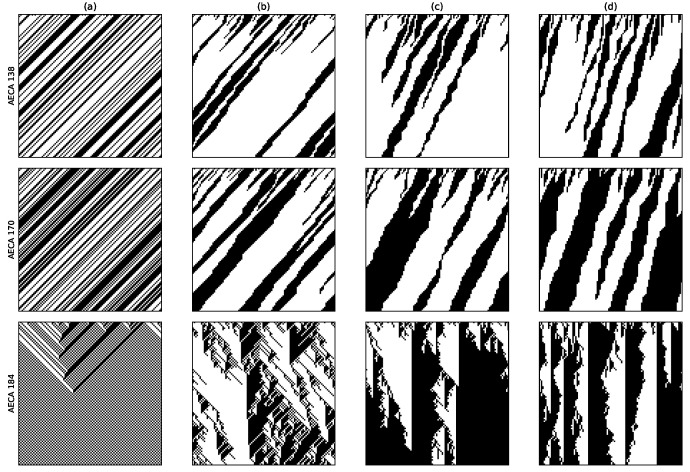
Time-space diagrams of AECA 138, 170 and 184 under various synchrony rates: (**a**) α=1, (**b**) α=0.8, (**c**) α=0.5 and (**d**) α=0.3. All initial configurations are the same, with $d_{ini}=0.5$. Time in each diagram flow from top to bottom.

**Figure 9:**
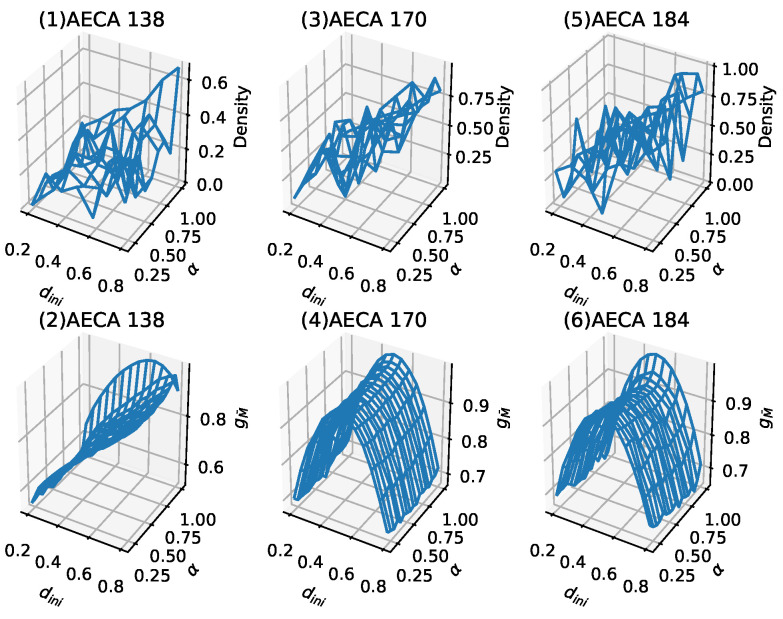
Comparison between the density-based sampling surface and the metric entropy-based sampling surface: the top (**1**), (**3**), (**5**) are the density-based sampling surfaces, the bottom (**2**), (**4**), (**6**) are sampling surfaces based on entropy. It can be verified that the entropy parameter makes the sampling surfaces no longer chaotic.

In the AECA classification experiments that uses asymptotic density as a parameter to study robustness, the results include a class of non-regular behavior [3], including AECA 138, 170 and 184 [8]. Figure 8 provides their time-space evolution diagrams, by which it is hard to distinguish the chaotic behavior along with the change of synchrony rate α. In contrast to the density parameter, all of these AECAs exhibit regular sampling surfaces measured in terms of the metric entropy $\bar{M}$ in Figure 9. Especially, all of the regular surfaces in Figure 9 can be regarded as functions of the initial density $d_{ini}$, because the change of synchrony rate α has little impact on them. Accordingly, this may allow some studies [25] on synchronous ECA 138, 170 and 184 to be available in the asynchronous counterparts.

According to the classification results in Table 1, AECA 138 belongs to Class 3 (c), while AECA 170 and 184 belong to Class 3 (a). The rules of Class 3 are all poorly robust, that is, they are sensitive to the initial condition $d_{ini}$ and synchrony rate α, whereby even a small change in $d_{ini}$ or α may cause large effects on the dynamical behavior.

## 5. Classification of Uncertainty Based on Kolmogorov–Sinai Entropy

The Kolmogorov–Sinai entropy defined by Equation (6) is used measure the uncertainty of forward evolutions of AECAs under a variety of initial condition $d_{ini}$ and synchrony rate α. Unlike the metric entropy $\bar{M}$, the KS entropy $H_{ks}$ has nothing to do with the density parameter by definition. For example, the AECA 204 is trivially deterministic such that its uncertainty is always zero whatever the values of $d_{ini}$ and α are, which can be exactly verified by the flat sampling surface in Figure 1c.

### 5.1. Results of Uncertainty-Based Classification

**Figure 10:**
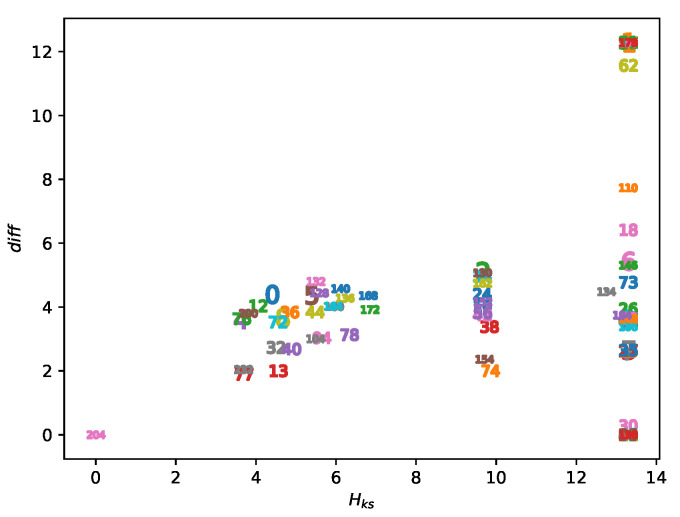
Plots of AECAs in accordance with the parameters $H_{ks}$ and diff.

According to Section 2.4 and Section 3.3, the KS entropy $H_{ks}$ can be approximated by $\max_{\alpha \in A,d_{ini}\in D}g_{H_{N}}$ where *N* is the maximal iterations of an AECA in each experiment (Algorithm 1). Similarly, let $diff=\max_{\alpha \in A,d_{ini}\in D}g_{H_{N}}-\min_{\alpha \in A,d_{ini}\in D}g_{H_{N}}$. Figure 10 provides the plots of $H_{ks}$ and diff of each AECA, which evidently allows a separation of these rules into three classes, as given in Table 2.

As shown in Figure 10, the uncertainty parameter $H_{ks}$ acts as the major influence factor to accomplish the divisions in Table 2. The degree of uncertainty of each class along with the distinct morphological features in their sampling surfaces are described below.

**Figure 11:**
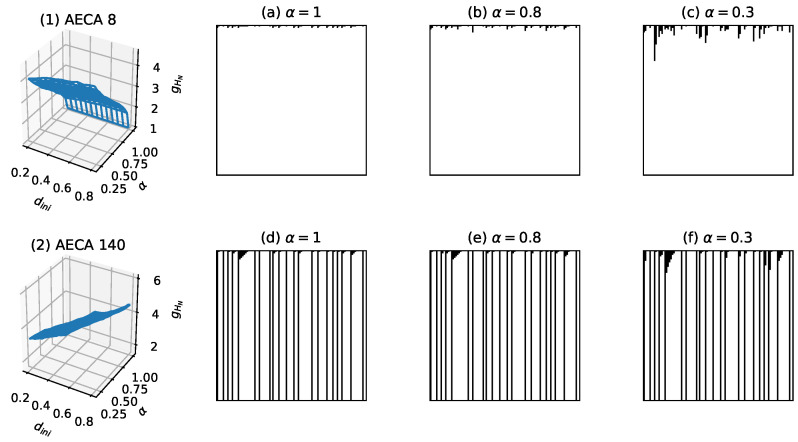
Sampling surfaces $g_{H_{N}}$ of (**1**) AECA 8 and (**2**) AECA 140 in Class I, along with their time-space diagrams under various synchrony rates: α=1, α=0.8 and α=0.3. Both rules satisfy $H_{ks}<8$ and show fast convergence in all time-space diagrams, giving rise to low uncertainty.

**Figure 12:**
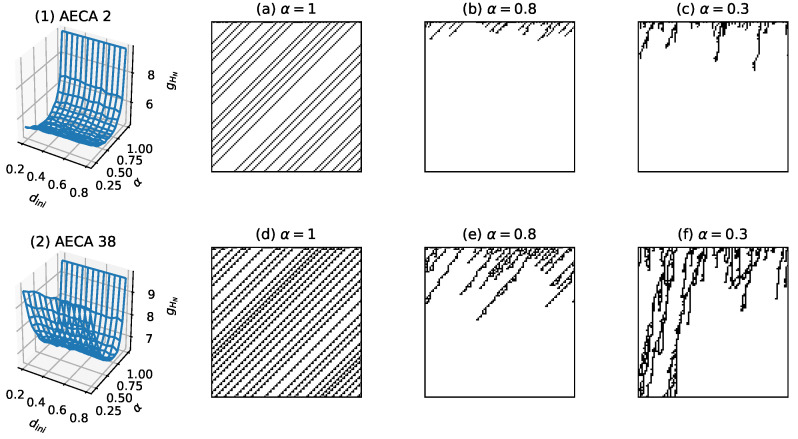
Sampling surfaces $g_{H_{N}}$ of (**1**) AECA 2 and (**2**) AECA 38 in Class II, along with their time-space diagrams under various synchrony rates: α=1, α=0.8 and α=0.3.

- Class I: All AECAs in Class I show fast convergence during the forward evolutions, thereby yielding low uncertainty $H_{ks}\in \left[0,8\right]$ (Figure 11). The surfaces $g_{H_{N}}$ of all rules resemble an inclined plane, and some rules may have mutations at α=1.
- Class II: All AECAs in Class II tend to converge but in a speed relatively slower than rules in Class I, resulting in moderate uncertainty $H_{ks}\in \left(8,12\right]$ (Figure 12). The surface $g_{H_{N}}$ of each rule approximates a non-linear continuous function of the asynchrony rate α that will reach the maximum at α=1. The initial density $d_{ini}$ has no effect on the $g_{H_{N}}$ in general.
- Class III: All AECAs in Class III are unable to converge till the end of experiments, thereby causing high uncertainty $H_{ks}>12$. The surfaces $g_{H_{N}}$ of all rules are either planar or non-linear functions of the synchronous rate α, and a minimum mutation occurs when α=1.

### 5.2. Details of Classification of Uncertainty

The AECAs in Class I can be divided into two sub-classes (a) and (b) according to whether the sampling surface is subject to a discontinuity at α=1. Sampling surfaces $g_{H_{N}}$ of AECAs in Class I(a) have a discontinuous mutation when α=1, which mainly depend on synchrony rate α and $d_{ini}$ has almost no influence. Examples of Class I(a) and Class I(b) are the AECA 8 and AECA 140, respectively, as shown in Figure 11. The AECA 140 also belongs to the so-called GAP Class [8].

The sampling surfaces $g_{H_{N}}$ of AECA 2 and 38 in Figure 12 are representatives of the sampling surfaces of all rules belonging to Class II. Both AECAs tend to converge in speeds that are slightly slower than the rules in Class I, resulting in a moderately higher level of uncertainty than the latters. In particular, although previous studies [21,22,23,24] proved the AECA 38 belonging to SPT class, its critical rate α=0.041 prevents the occurrence of abrupt transition of dynamical phases during our experiments due to the protocol in Section 3.2. As a result, the sampling surface $g_{H_{N}}$ of AECA 38 in Figure 12 possibly indicates a moderate uncertainty when the rule evolves in the active phase, i.e., α∈(0.041,1].

**Figure 13:**
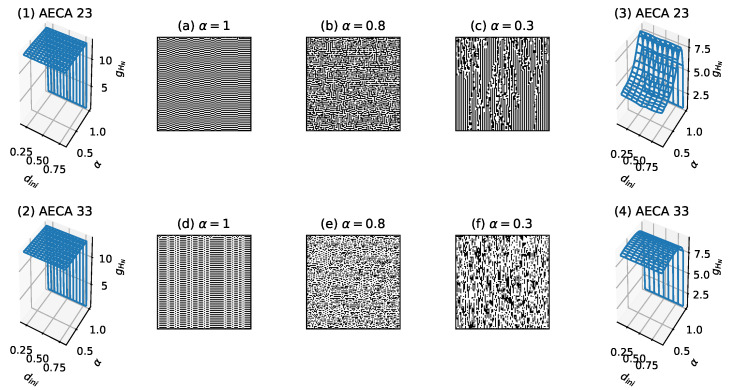
Sampling surfaces $g_{H_{N}}$ of (**1**) AECA 23 and (**2**) AECA 33 along with the time-space diagrams under different synchrony rates: α=1, α=0.8 and α=0.3. Sampling surfaces $g_{H_{N}}$ of (**3**) AECA 23 and (**4**) AECA 33 measured using modified parameters: L=10 and N=3000.

Moreover, the Class III in Table 2 consists of the rest of AECAs other than the rules classified into Class I and Class II, of which the uncertainty $H_{ks}$ seems difficult to be estimated accurately in the time range designated in Section 3.3, due to the complex and non-convergent behavior which, in general, requires an exponential growth in time to measure as the length of cell ring increases. For example, experimental results in Figure 13(1,2) show that the AECA 23 and AECA 33 respectively take high uncertainty, i.e., $H_{ks}>12$, and their forward evolutions never converge in the time range specified by the parameter *N* = 10,000. Fortunately, these AECAs are not sensitive to the initial condition $d_{ini}$, whereby it is possible to reduce the length of cell ring *L* so as to enable more accurate evaluation of their uncertainty $H_{ks}$ (sampling surface $g_{H_{N}}$) in a reasonable time scale. As a result, Figure 13(3,4) illustrate the sampling surfaces of AECA 23 and AECA 33, respectively, that are estimated by experiments after decreasing the ring length *L* and maximal iteration time *N* to 10 and 3000, respectively.

The above scheme can carry over to all other rules in Class III that are insensitive to the initial density $d_{ini}$, similar to the AECA 23 and AECA 33. This allows to divide them into two subclasses Class III (a) and Class III (b), as given in Table 2. Specifically, the sampling surfaces of rules belonging to Class III (a) look like a plane with a discontinuous mutation occurring at α=1 (see Figure 13(4)). Likewise, the sampling surfaces of rules belonging to Class III (b) resemble a non-linear and continuous function of synchrony rate α with a discontinuous mutation at α=1 (see Figure 13(3)).

**Figure 14:**
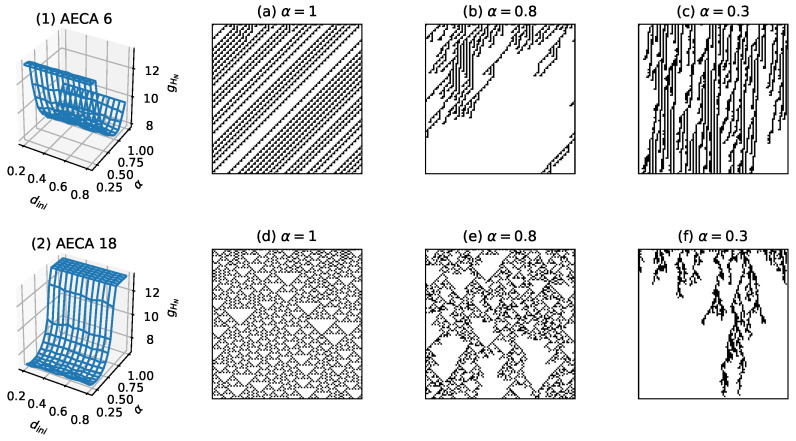
Sampling surfaces $g_{H_{N}}$ of (**1**) AECA 6 and (**2**) AECA 18 belonging to Class III (c), along with the time-space diagrams under different synchrony rates: α=1, α=0.8 and α=0.3.

For AECAs belonging to Class III (c) and Class III (d), however, the reduction of the length of cell ring seems unavailable for measuring the uncertainty because of their crucial sensitivity to the initial condition $d_{ini}$. In such a situation, the Class III (c) comprises all AECAs that belong to the SPT class [21,22,23,24]. Thus, the sampling surface of an AECA will grow rapidly to reach the maximum in the active phase of synchrony rates, while dropping the surface to much low level in the passive phase. For example, the AECA 18 has critical rate at 0.714 with the active phase consisting in the interval α∈[0.714,1]. As shown in Figure 14(2), the rule exhibits distinct dynamical behavior in different phases that indicates a high degree of uncertainty, with the maximal value of surface $g_{H_{N}}$ appearing in α>0.75. The case for the AECA 6 in Figure 14(1) is similar.

**Figure 15:**
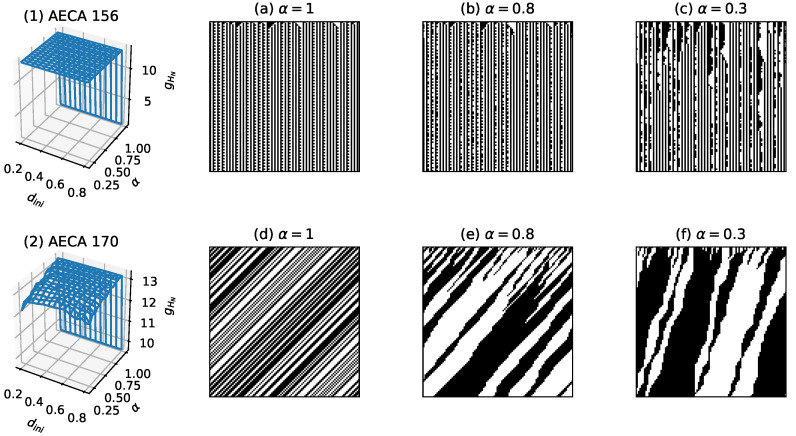
Sampling surfaces $g_{H_{N}}$ of (**1**) AECA 156 and (**2**) AECA 170, together with the time-space diagrams under various synchronous rates: α=1, α=0.8 and α=0.3. Both sampling surfaces almost reach the plane $g_{H_{N}}$ = log(10,000) = 13.8.

Furthermore, Class III (d) contains those extra AECAs that are basically $d_{ini}$ sensitive, and have their entire surface $g_{H_{N}}$ almost reach the upper bound log(N) as illustrated in Figure 15, which imply an unbounded increase of the uncertainty as the number of time steps to iterate the rules approaches the infinity (Equation (6)). For this reason, we denote the uncertainty of AECAs in Class III (d) as unbounded in Table 2.

## 6. Conclusions

In order to fully distinguish the dynamical characteristics of the elementary class of CAs under asynchronous updating, this paper proposed two types of entropies, with one devoted to measure the robustness of AECAs against the randomness due to α-asynchronism, and another one used for estimating the uncertainty in the forward evolutions. The robustness of each AECA was measured in terms of a metric entropy which expresses the asymptotic mean entropy of local patterns. Numerical experiments showed that it is capable of classifying every AECA into one of four classes in accordance with its stability in response to the variations of initial condition and synchrony rate. Especially, the results given in Table 1 are basically consistent with the density-based classification [8], demonstrating the effectiveness of our metric entropy-based estimation of robustness. On the other hand, the entropy-based measure succeeded in classifying all ECAs, i.e., the 88 representative rules, and allow further division of each class into several sub-classes according to the morphological features in the sampling surfaces. Especially, for some synchronous ECAs, the change of the length of initial configurations will lead to the emergence of phase transition in their evolutions [26]. Similar phenomena may be found under asynchronous updating, like the AECA 6 and AECA 50, which show phase transition due to the variation of synchrony rate α.

As with the topological entropy for synchronous ECAs [12], this paper attempted to define a Kolmogorov–Sinai entropy over the entire time-space evolution diagrams of each AECA, by which the rule can be classified into one of three groups according to its uncertainty about the initial condition and asynchrony rates. Roughly speaking, the first and second classes comprise those simple AECAs that will eventually converge within the experimental time limit, and the major difference between them is the speed of convergence. This is conformable to a natural and intuitive observation: faster convergence, lower uncertainty. For the third class, however, all AECAs are complex and thus reveal non-convergence in the forward evolutions within the time limit of experiments, indicating high degree of uncertainty that may not be measured accurately by the current protocol. Despite a lack of enough accuracy, we made efforts to modify the experimental protocol and combine with other results [21,22,23,24], which enables a preliminary division of all complex AECAs into four sub-classes according to their distinct features of sampling surfaces. Nevertheless, how to design a more efficient protocol for accurate measure of KS entropy and how to achieve a more reliable and sophisticated classification of all complex AECAs, will be our essential work in the future. 

## Figures and Tables

**Table 1:** Robustness classification of asynchronous elementary cellular automata based on metric entropy $\bar{M}$.

Class	Sub-Class	Robustness	Rules
Class 1	(a)	Stable	1,3,5,7,11,14,15,19,23,27,29,33,35,43,51,142
	(b)		9,13,22,25,28,30,37,41,45,54,57,60,62,73,77,78,90,94, 105,110,122,126,150,156
Class 2		Stable	0,2,8,10,24,34,42,56,74,130,152,154,162
Class 3	(a)	Unstable	132,140,170,184,200,204,232
	(b)		4,12,36,44,72,76,104,108,164,172
	(c)		32,40,128,136,138,160,168
Class 4		Unstable	6,18,26,38,46,50,58,106,134,146,178

**Table 2:** Classification of AECAs based on estimated uncertainty $H_{ks}$.

Class	Sub-Class	Uncertainty	Rules
Class I	(a)	Low	0,4,5,8,12,13,32,36,40,44,72,76,77,78,94,104,200,204,232
	(b)		128,132,136,140,168,172
Class II		Medium	2,10,24,34,38,42,56,74,130,152,154,162
Class III	(a)	High	1,3,9,11,19,22,25,27,33,35,37,41,43,51,54,57,60,62,105,110,122,142
	(b)		7,14,15,23,30,45,90,126,150
	(c)		6,18,26,50,58,106,134,146,178
	(d)	Unbounded	28,29,73,156,108,138,170,184

## Data Availability

The source programs and experimental data could be found at the following site https://github.com/tanlei0/ECA-classifcation-based-on-entropy.
